# Gallbladder fossa lymphangioma encasing the common bile duct: a case report and review of the literature

**DOI:** 10.1097/MS9.0000000000002083

**Published:** 2024-04-24

**Authors:** Pirouz Samidoust, Maziar Moayerifar, Mahboobeh Gholipour, Selvana Poursadrolah, Athar Zamani, Mani Moayerifar

**Affiliations:** a Razi Clinical Research Development Unit; b Department of Vascular Surgery, Razi Hospital; c Department of Pathology and Laboratory Medicine; d Student Research Committee, School of Medicine; e Department of Cardiology, Healthy Heart Research Center, Heshmat Hospital, School of Medicine, Guilan University of Medical Sciences, Rasht, Iran

**Keywords:** common bile duct, cystic, gallbladder, gallbladder fossa, lymphangioma

## Abstract

**Introduction:**

Lymphangiomas are rare low-flow lymphatic vessel malformations mostly diagnosed in childhood. Among the abdominal lymphangiomas, the gallbladder lymphangioma is a rare presentation, and only a few cases have been reported till date, of which majority were cystic lymphangiomas.

**Case presentation:**

The authors present a case of a 48-year-old female with abdominal pain and normal physical examination and laboratory findings. On the performed imaging, a multiloculated cystic lesion, located in the gallbladder fossa, was found. The patient underwent open cholecystectomy, and surprisingly, the cyst extended to the common bile duct, which was separated cautiously. The cyst was resected, and the histopathological findings confirmed cystic lymphangioma.

**Conclusion:**

The cystic lymphangioma of the gallbladder can be asymptomatic, although most of the literature has reported abdominal pain. The diagnosis of lymphangioma is complicated, especially when intra-cystic hemorrhage happens. Ultrasonography, computed tomography, and magnetic resonance imaging are usually performed. The surgical excision of the cyst and the gallbladder is the treatment of choice. Although bile duct involvement is extremely rare, it should be considered during the surgery.

## Introduction

Lymphangioma, with an incidence rate of 1:2000–4000 in children, is a common benign lymphatic vessel lesion in childhood, affecting both genders equally. Most of the cases are diagnosed before the age of two, and the presentation of the lesion in adulthood is rare. Lymphangioma is commonly located in the head and neck (70–80%) but can present in rarer sites, including the lungs, pleura, pericardium, and gastrointestinal tract. Among intraabdominal lesions, gallbladder lymphangioma is extremely rare and usually overlooked until it grows to a large mass, resulting in abdominal discomfort^1–3^. We represent a large cystic lymphangioma of gallbladder fossa encasing the common bile duct (CBD) and the gallbladder.

## Case presentation

**Figure 1:**
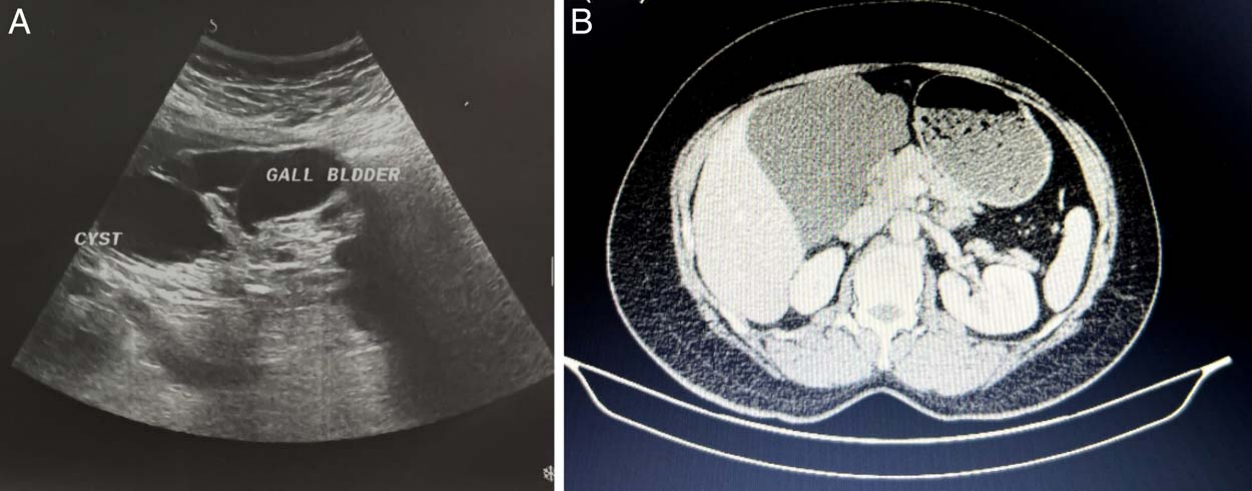
(A) A large multilobulated cystic lesion in ultrasonography is shown. (B) Abdominal computed tomography demonstrates a large lobulated non-enhancing wall cystic lesion in gallbladder fossa.

**Figure 2:**
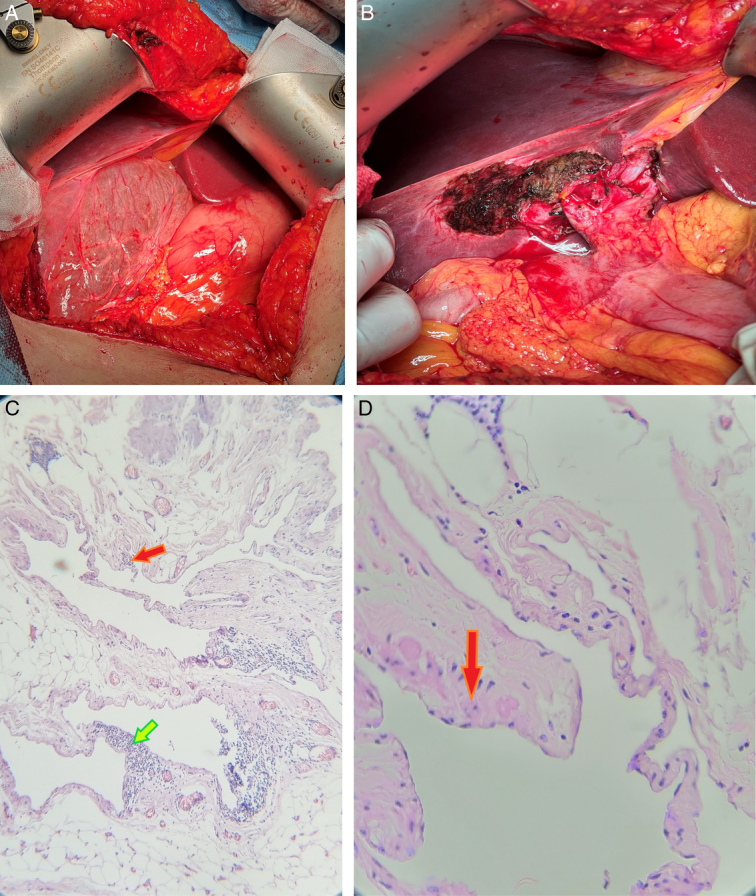
(A) A large cystic lymphangioma encasing the gallbladder is shown. (B) Intraoperative picture taken following mass resection, and anastomosis of the common bile duct. (C) The red arrow shows thin-walled, dilated lymphatic channels without intraluminal proteinaceous material and lymphocytes. The green arrow points to lymphocyte aggregation. (D) Bland, flat endothelial cells are marked.

A 48-year-old female was referred to our department complaining of intermittent abdominal pain for 2 years. The non-positional pain was colicky, with gradual onset limited to the epigastric area and the right upper quadrant, with no relevance to defecation, eating, and breathing. There were no associated symptoms. The patient had a history of hypertension herself and hepatic cancer in her father. She has been taking oral contraceptives for the past 10 years. A month before our visit, an endoscopy and rapid urease test were performed, and no abnormal findings were revealed. On physical examination, the patient was afebrile, with normal vital parameters. In the laboratory results, there was no abnormal finding. The ultrasonography (US), obtained right after the presentation, revealed a large, well-circumscribed multilocular cystic mass with dimensions of 10.6 × 9.2 × 12.4 cm, having multiple, thin septa and no solid component located in the gallbladder fossa (Fig. 1). Suspecting a hydatid cyst, we measured an anti-hydatid antibody, and the result was negative. A week later, the computed tomography (CT) with contrast demonstrated a normal gallbladder. A large cystic lesion with scalloping borders measuring approximately 11.6 × 9 × 11 cm (550 cc) in the gallbladder fossa with left lateral extension up to the left subhepatic space and inferior expansion to level of the D2 segment of the duodenum was seen (Fig. 1). No typical feature of hydatid cyst was detected, and the cyst had completely encased the gallbladder and a tiny branch of the proper hepatic artery. Based on the findings, the cyst was diagnosed as a gallbladder lymphangioma. Without further imaging, after a few days, the patient underwent an open cholecystectomy with a Chevron incision. Opening the abdomen, the large cyst filled with fluid was seen, lying beneath the liver adherent to the left liver lobe, stomach, and gastrohepatic and hepatoduodenal ligaments (Fig. 2). The gallbladder and the CBD were encased by the cyst. The cyst and gallbladder were both successfully resected. Due to adhesion, the CBD was cut; an end-to-end anastomosis of the CBD was created without any complications, and a drain was inserted. No evidence of recurrence on the third month follow-up was noted. Microscopic sections of the received specimen demonstrated small to large caliber vascular spaces, lined by endothelial cells, which were filled with eosinophilic proteinaceous fluid and intraluminal clusters of lymphocytes (Fig. 2). Cytological examination of cyst-like drained fluid revealed numerous lymphocytes, and the diagnosis of cystic lymphangioma was confirmed.

## Discussion

Lymphangioma is an infrequent nonmalignant malformation of the lymphatic vessels. This lesion is categorized as simple, cavernous, and cystic types based on the histological characteristics. The simple type is mostly located on skin, while the cavernous and cystic types are mainly deep in the body. In contrast to cystic type, in cavernous lymphangioma, there is a connection between dilated lymphatic spaces and the normal adjacent lymphatic vessels. Abdominal lymphangiomas can be seen uncommonly, and among them, gallbladder lymphangioma is very rare^2–4^. There are several theories corresponding to the etiology of these lesions, indicating the role of external factors like abdominal surgery, trauma, radiation, and any other cause that can interrupt the normal lymphatic drainage^4^.

A PubMed search using the MeSH terms “Lymphangioma” and “Gallbladder” was conducted. We studied the articles reporting cases of cystic or cavernous lymphangioma of gallbladder regardless of age, sex, and presence of the symptoms. The inclusion criteria were case reports written in English presenting patients admitted with gallbladder lymphangioma. Papers in languages other than English and article types other than case reports were excluded. The final analysis included 15 articles published between 1995 and 2023.

Although early studies have not reported any predisposing factors in the family history or past medical history, our case had a history of hepatic cancer in her father, and the probable correlation is still unstudied. Reported cases of gallbladder lymphangioma are mainly women, and the age varies from 2 to 66 (Table 1)^3–17^. Although lymphangioma can be an incidental finding^4–6,9,13,15^, at the time of presentation, most of the cases had symptoms including chronic or acute right upper quadrant pain as a result of the compression effect of the cyst^3,8,10,12,14–17^. Intra-cystic hemorrhage occurred in some cases and made the lesion harder to diagnose^4,6,12^, while no rupture of the cyst is reported. Diagnosing gallbladder lymphangioma is mostly based on imaging, and the laboratory results and abdominal examination were normal in most of the cases. While US shows a simple or multilocular mass filled with anechoic fluid, complementary CT demonstrates a cystic lesion with the same density as water and uniform septal thickness^9^. Magnetic resonance imaging (MRI), magnetic resonance cholangiopancreatography (MRCP), and endoscopic retrograde cholangiopancreatography (ERCP) are widely used to receive additional information about the lesion vicinities and communication to the biliary tract. In an MRI performed in the axial and coronal planes, typical findings include a simple or multiloculated cystic lesion without any solid component, and in some cases, the septa of the cyst are enhanced^9,14^. MRI is also helpful to differentiate between intra-cystic solid mass and intra-cystic hemorrhage, while CT may be less efficient if the case is initially presented after hemorrhage^4^. In a particular case in addition to the imaging mentioned above, an abdominal angiography was performed preoperatively showing avascular cystic lesion receiving a branch of cystic artery^5^. The multiseptated cystic mass originating from the gallbladder fossa can replace, encase^8,^ or encapsulate^4^ the gallbladder. Similar to our case in which the CBD was encased by the lesion, a previous study reported a gallbladder lymphangioma encasing the entire extra-hepatic bile ducts^8^. Considering all the available imaging, gallbladder lymphangioma can be misdiagnosed due to its rare presentation and possible complications, namely intra-cystic hemorrhage or inflammation. A number of authors have indicated simultaneous lymphangioma and microlithiasis^10^, asymptomatic cholelithiasis^9,^ or acute cholecystitis^12,14,^ which made the diagnosis more complicated. The cyst was also misdiagnosed as hydatid cyst. To overcome the diagnostic problems, five out of 16 cases underwent exploratory laparotomy^3,8,9,13,15^. All early studies have suggested laparoscopic or open resection of the mass with the gallbladder as the treatment of choice in gallbladder lymphangioma, and no conservative management has been reported. In one study mentioned earlier, due to bile duct involvement, the extra-hepatic bile ducts were also resected en bloc, and Roux-en-Y choledochojejunostomy was done^8^. None of the authors acknowledged any recurrence after surgery. Histopathology examination of resected masses confirmed cystic lymphangioma of the gallbladder in 14 out of 16 cases, and the two remainder were acknowledged as the cavernous type. The microscopic findings in a cystic lymphangioma include presence of lymphocytes, variable lymphatic spaces lined by flat endothelium, and smooth muscles in cyst walls, while in the cavernous lymphangioma, no smooth muscle is seen^5,16^. Only few works in literature mentioned using immunohistochemistry tests such as D2-40 staining^10,14^.

**Table 1:** Summary of gallbladder lymphangioma cases presented in the literature.

Author and year of publication	Type	Age and sex	Clinical presentation	Size	Histopathology	Imaging	Lab findings	Bile duct preserved	Management	Follow-up
Ohba *et al*.^5^, 1995	Cystic	44, female	N/A	20 × 18 × 15 cm	Presence of small lymphatic spaces lined with a flat endothelium, lymphoid tissue, and smooth muscle in the cyst wall	US, CT, MRI, ERCP, abdominal angiography	Hemoglobin = 10.5 g/dl	Yes	Open cholecystectomy	N/A
Choi *et al*.^6^, 2002	Cavernous	54, male	RUQ pain	6 × 3 × 2 cm	Lymphatic vessels lined by flat endothelium in the subserosal connective tissue layer of the gallbladder	US, CT, MRI, MRCP, ERCP	Elevated blood glucose	Yes	Open cholecystectomy (patient underwent cholecystectomy after intra-cystic hemorrhage)	N/A
Yang *et al*.^7^, 2003	Cystic	28, female	Abdominal pain	15 × 10 cm	N/A	US, CT, MRI	N/A	N/A	Laparoscopic cholecystectomy	N/A
	Cystic	36, female	Abdominal pain	10 × 18 × 5 cm	N/A	US, CT, MRI	N/A	N/A	Laparoscopic cholecystectomy	N/A
Noh *et al*.^8^, 2005	Cystic	29, female	RUQ pain, nausea, and vomiting	N/A	N/A	US, MRI, MRCP, ERCP	Normal	Adhere to the entire extra-hepatic bile duct	Cholecystectomy exploratory laparotomy	No evidence of recurrence in 9 months
Kim *et al*.^4^, 2007	Cystic	60, male	No symptoms	5.5 × 4.3 × 3.5 cm	Multiple lymphocyte aggregations	US, CT, MRI, MRCP	Normal	Yes	Open cholecystectomy	CT showed no evidence of recurrence in 12 months
Shikano *et al*.^9^, 2007	Cystic	66, female	No symptoms	6 × 3 × 2 cm	Small lymphatic spaces lined with flat endothelium, lymphoid tissue, and smooth muscle in cyst wall	US, CT, MRI, MRCP	Cancer antigen 19-9 = 42	Yes	Extended cholecystectomy, including the cystic mass and 5 mm of the liver margin	N/A
Woo *et al*.^10^, 2007	Cystic	34, female	Sudden RUQ and fever	5 × 3 × 2 cm	Variable-sized clear spaces that were lined by flat endothelium with some lymphoid tissue in the wall	US, CT, ERCP	Abnormal liver tests	Yes	Laparoscopic cholecystectomy	No evidence of recurrence at 6 months follow-up
Stunell *et al*.^11^, 2007	Cystic	30, female	Abdominal pain	15.2 × 9.4 cm	Multiple cystic spaces lined by a layer of cells morphologically consistent with endothelium	US, CT, MRCP	Erythrocyte sedimentation rate = 27 C-reactive protein = 24.7	N/A	En bloc excision of gallbladder and the cyst	N/A
Bridda *et al*.^12^, 2011	Cystic	17, male	Fever, nausea, vomiting, and RUQ pain radiating to right shoulder	10 × 5 cm	Lymphatic vessel lined by flat endothelium in the subserosal connective tissue layer of the gallbladder	US, CT	Leukocytosis and mild elevated aspartate aminotransferase	Yes	Laparoscopic cholecystectomy (Preoperation diagnosis was acute acalculous cholecystitis complicated by subhepatic abscess while the final diagnosis got to be cystic and hemorrhagic lymphangioma.)	No evidence of recurrence at 3-month follow-up
Han *et al*.^13^, 2011	Cystic	2-year-6-month-old, male	No symptoms	5.3 × 3.7 × 3.8 cm	Pathological examination gave the diagnosis as lymphangioma of the gallbladder wall	US, CT	Normal	N/A	Cholecystectomy during exploratory laparotomy (The preoperative diagnosis was neoplasm of the gallbladder.)	No ultrasonic evidence of recurrence
Boskovski *et al*.^14^, 2012	Cystic	26, male	RUQ pain and diarrhea	8.50 × 1.70 × 1.5 cm	Widely patent, anastomosing vascular channel lined by flattened epithelium and supported by thin, fibrofatty septa	US, CT, MRI	Normal	N/A	Open cholecystectomy en bloc	N/A
Nazarewski *et al*.^15^, 2003	Cystic	41, female	No symptoms	13 × 5.5 × 6.5 cm	A thin-walled multilocular lesion corresponding to a lymphangioma	US, CT, MRI	Normal	Yes	Open cholecystectomy	N/A
Vu *et al*.^16^, 2019	Cavernous	42, female	Intermittent RUQ pain	7.9 × 5.7 × 4.2 cm	Numerous irregular ectatic vascular spaces and mature lymphocytes	US, CT, MRI	Normal	Yes	Laparoscopic cholecystectomy and intraoperative cholangiogram	Resolution of abdominal symptoms
Dukmak *et al*.^17^, 2022	Cystic	14, female	RUQ pain	12 × 8 cm	Thin-walled cyst composed of many dilated lymphatic spaces	CT	Borderline bilirubin	Yes	Open cholecystectomy	N/A
Garg and Gupta^3^, 2023	Cystic	56, female	RUQ pain	2.5 × 1.5 × 6 cm	N/A	US, CT, MRI, MRCP	Normal	Yes	Mass resected en bloc with the gallbladder during exploratory laparotomy (The preoperative diagnosis was a hydatid cyst, and the patient was administered albendazole for 1 month)	N/A

CT, computed tomography; ERCP, endoscopic retrograde cholangiopancreatography; MRCP, magnetic resonance cholangiopancreatography; MRI, magnetic resonance imaging; RUQ, right upper quadrant; US, ultrasonography.

## Conclusion

Despite the fact that gallbladder lymphangioma is rare, it is considerable in patients with right upper quadrant (RUQ) pain, tenderness, palpable mass, and associated symptoms. US, CT, and MRI are usually used to assess the suspected lesion. However, MRCP and ERCP may be essential for a close observation on biliary tree. The gold standard treatment is the excision of the cyst and gallbladder. As experienced in our case, the CBD may be involved, which should be preserved delicately.

### Patient perspective

On follow-up sessions, she was totally satisfied with the result of the treatment. The patient experienced a complete remission with no recurrence of symptoms or any complaints related to the surgery.

## Ethical approval

Ethical approval for this study was provided by the Research Ethics Committees of Guilan University of Medical Sciences, Rasht, Iran, on 10 January 2024. The approval ID is IR.GUMS.REC.1402.515.

## Consent

Written informed consent was obtained from the patient for the publication of this case report and accompanying images. A copy of the written consent is available for review by the Editor-in-Chief of this journal on request.

## Sources of funding

There was no funding, financial support, and sponsorship for this article.

## Author contribution

P.S., Maziar M., M.G., S.P., A.Z., and Mani M.: all contributed to this project by designing the study, collecting the data, drafting, and revising the work. All authors agreed to be accountable for all aspects of the work, ensuring that questions related to the accuracy or integrity of any part of the work are investigated and resolved.

## Conflicts of interest disclosure

There are no conflicts of interest.

## Research registration unique identifying number (UIN)

This paper is a case report, and there is no registration.

## Guarantor

Dr Pirouz Samidoust and Dr Mani Moayerifar.

## Data availability statement

Not applicable to this article.

## Provenance and peer review

Not commissioned, externally peer-reviewed.
